# Green Synthesis of Hierarchical Metal–Organic Framework/Wood Functional Composites with Superior Mechanical Properties

**DOI:** 10.1002/advs.201902897

**Published:** 2020-02-06

**Authors:** Kunkun Tu, Begoña Puértolas, Maria Adobes‐Vidal, Yaru Wang, Jianguo Sun, Jacqueline Traber, Ingo Burgert, Javier Pérez‐Ramírez, Tobias Keplinger

**Affiliations:** ^1^ Wood Materials Science Institute for Building Materials ETH Zürich Zürich 8093 Switzerland; ^2^ WoodTec Group Cellulose & Wood Materials EMPA Dübendorf 8600 Switzerland; ^3^ Institute for Chemical and Bioengineering Department of Chemistry and Applied Biosciences ETH Zurich Zurich 8093 Switzerland; ^4^ Eawag Swiss Federal Institute of Aquatic Science and Technology Dübendorf 8600 Switzerland

**Keywords:** green synthesis, hierarchical porosity, mechanical properties, metal–organic frameworks, wood‐based composites

## Abstract

The applicability of advanced composite materials with hierarchical structure that conjugate metal–organic frameworks (MOFs) with macroporous materials is commonly limited by their inferior mechanical properties. Here, a universal green synthesis method for the in situ growth of MOF nanocrystals within wood substrates is introduced. Nucleation sites for different types of MOFs are readily created by a sodium hydroxide treatment, which is demonstrated to be broadly applicable to different wood species. The resulting MOF/wood composite exhibits hierarchical porosity with 130 times larger specific surface area compared to native wood. Assessment of the CO_2_ adsorption capacity demonstrates the efficient utilization of the MOF loading along with similar adsorption ability to that of pure MOF. Compression and tensile tests reveal superior mechanical properties, which surpass those obtained for polymer substrates. The functionalization strategy offers a stable, sustainable, and scalable platform for the fabrication of multifunctional MOF/wood‐derived composites with potential applications in environmental‐ and energy‐related fields.

## Graphical abstract

A versatile and sustainable synthesis protocol is developed for the synthesis of a series of metal–organic frameworks (MOF)/wood composites. Nucleation sites for the in situ MOF growing within the wood structure are readily created by a sodium hydroxide treatment resulting in a material that exhibits hierarchical porosity and superior mechanical properties, which surpass those obtained over polymer substrates.

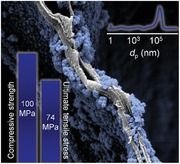

## 1 Introduction

Metal–organic frameworks (MOFs) are crystalline coordination polymers with tunable porosity that are composed of 3D networks of metal ions (or clusters) and organic linkers. Their unique micro/mesopore architecture results in high specific surface area, large porosity, low density, and structural diversity,^1^ which makes them highly attractive candidates for numerous applications, including gas storage and separation,^2^ catalysis,^3^ drug delivery,^4^ sensing,^5^ and energy storage.^6^ However, due to the crystalline nature of MOFs, they are most commonly found as powders, thus, their processability and handling remain a significant challenge.^7^ Integrating MOFs onto or into various substrates to produce shapeable and cost‐efficient materials constitutes one way to expand the potential applications of these functional materials. In this regard, MOFs have been deposited or grown on various polymer substrates^8^ by means of direct mixing,^9^ in situ growth,^10^ layer‐by‐layer,^11^ and continuous flow synthesis^12^ resulting in composite materials that exhibit a complex multilevel network of macropores, mesopores, and micropores, in which the macro‐ and mesoporous structures enhance the diffusion kinetics and accessibility to the active sites located in the micropores of MOFs.^13^

A common way to fabricate porous MOF‐containing composites are bottom‐up methods such as freeze‐casting,^14^ freeze‐drying,^15^ solvent‐casting,^16^ or electrospinning.[qv: 9a] While these methods lead to composites with sufficient porosity, they are often characterized by relatively weak mechanical properties,^17^ due to the well‐known trade‐off between high porosity and satisfying mechanical properties, which substantially limits their applicability in pressure‐driven gas or liquid adsorption and separation processes.^18^ Even though various methods have been proposed to improve the mechanical properties, i.e., increasing the polymer‐volume fraction within the composites,^19^ cross‐linking of the polymer^20^ as well as the incorporation of additional reinforcing fibers,^21^ the resulting composites can still be limited by MOF aggregation, weak interaction between the substrate and MOFs, or low permeability. Furthermore, the flexibility and swelling of the polymer membrane may result in the segregation of the MOF layer from the membrane surface.^22^

Hence, finding an alternative substrate that provides both sufficient porosity and excellent mechanical properties is a prerequisite.^23^ A range of biological materials that fulfills these requirements is available in nature, among which wood outstands as a promising example. Wood is a hierarchically‐structured material across several length scales composed of well‐connected hollow fibrous structures.^24^ This unique hierarchical and open porous structure with superior mechanical properties along with its light weight offers an ideal scaffold to manufacture high‐performance composite materials, such as transparent wood,^25^ oil/water separation membranes,^26^ solar steam generation devices,^27^ mechanically tunable wood,^28^ and stimuli responsive wood.^29^ Additionally, wood exhibits abundant hydroxyl groups that can act as active sites for chemical modifications facilitating the in situ functionalization with polymers or inorganic materials leading to advanced functionality.^30^

Wood scaffolds represent a green alternative compared to synthetic polymer systems, which often include time‐ and energy consuming processes to build up the needed 3D porous structure. However, studies that exploit all these beneficial aspects to develop MOF/wood composites are scarce.^31^

**Figure 1:**
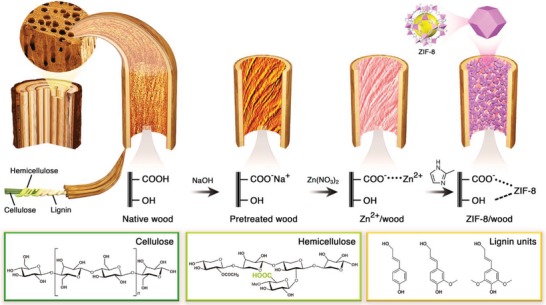
Schematic representation of the fabrication process to obtain ZIF‐8/wood composites.

Here, we report a versatile and simple strategy for the sustainable synthesis of composites with unique hierarchical structure and superior mechanical properties, based on the in situ growth of different types of MOFs within diverse wood substrates (**Figure** **1**). While MOF provides the micro‐ and mesoporous network, macroporous wood serves as mechanical support, opening up the avenue for a new type of composite materials.

## 2 Results and Discussion

**Figure 2:**
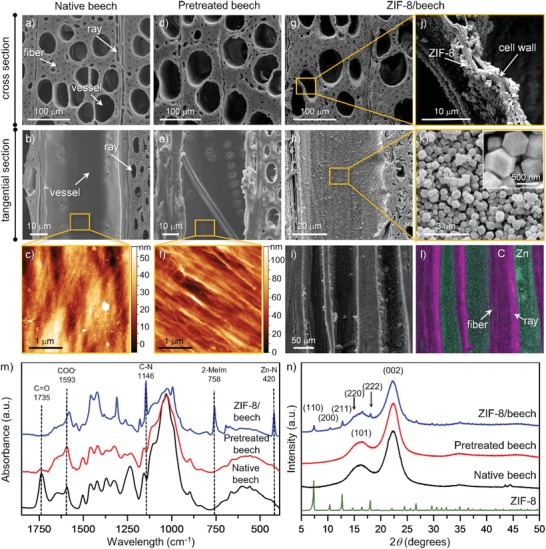
Cross‐sectional (first line) and tangential‐sectional (second line) SEM images of a,b) native beech, d,e) pretreated beech and g–k) ZIF‐8/beech composite. The inset in (k) corresponds to the magnified SEM image of ZIF‐8 nanocrystals in ZIF‐8/beech composite. AFM images of the cell wall layer facing to the cell lumen of c) native beech and f) pretreated beech. i) SEM image and l) corresponding elemental maps of zinc (green) and carbon (pink) for the ZIF‐8/beech composite. m) FTIR spectra of native beech, pretreated beech and ZIF‐8/beech composite. n) XRD patterns of native beech, pretreated beech, ZIF‐8/beech composite and pure ZIF‐8.

Beech, a widely used diffuse‐porous hardwood species, mainly composed of tubular cells, i.e., vessels and fibers along the longitudinal direction, whose cell walls consist of cellulose fibrils embedded in a matrix of hemicelluloses and lignin (**Figure** **2**a,b), was selected to prepare the initial composite material. First, we performed a simple and effective sodium hydroxide (NaOH) solution pretreatment of the wood samples. This step fulfills simultaneously two main functions: (i) it ensures the ion exchange of the proton in the wood inherent carboxyl groups by sodium cations, providing the nucleation sites for the subsequent growing of the MOF structure, and (ii) it provides a rough fibrillar structure that boosts the anchoring of the MOFs to the wooden substrate. Fourier‐transform infrared (ATR‐FTIR) spectra confirmed the successful chemical transformations that occurred during this NaOH pretreatment process (Figure 2m). The decrease in the intensity of the carbonyl group (—C=O) at 1735 cm^−1^ and the increase of carboxylate groups (—COO^−^) at 1593 cm^−1^ for the pretreated beech indicate the partial removal of hemicelluloses and the successful transformation from —COOH to —COONa after the alkaline treatment. Hence, the negative surface charge of bulk beech samples measured by zeta potential analysis increased by the pretreatment from −12.05±1.87 mV to −38.44±1.99 mV, leading to strong affinity to metal cations (Table S1, Supporting Information). Atomic force microscopy (AFM) revealed that the NaOH pretreatment also removed the innermost surface layer of the cell walls facing to the empty lumen of the cells (Figure 2c) and exposed the underlying cellulose microfibril structure (Figure 2f). This surface layer is composed of hemicelluloses (mannan and xylan) and lignin‐like polyphenols.^32^ The decrease of the IR‐peaks at 1396 cm^−1^ attributed to C—H deformation in hemicellulose and 1234 cm^−1^ corresponding to C—O stretch in lignin demonstrated the partial removal of hemicellulose and lignin. Note that the X‐ray diffraction (XRD) pattern confirmed that the NaOH pretreatment does not alter cellulose as its initial crystal structure remains intact (Figure 2n). After pretreatment, the beech samples were thoroughly washed with water until the pH value of the washing water equaled 9. In order to retain the porous structure, the samples were kept in wet state after washing, as conventional vacuum drying of pretreated beech leads to pronounced cell wall shrinkage accompanied by the blockage of the lumina (Figure S1, Supporting Information), which would hamper the subsequent growth of the MOF. To fix the original pretreated beech structure in the dry state for scanning electron microscopy (SEM) and AFM analysis, the samples were subjected to a freeze‐drying process to avoid shrinkage (Figure S2, Supporting Information).

The next step consists of the in situ formation of the MOF, i.e., zeolitic imidazolate framework‐8 (ZIF‐8) in this case, crystal nuclei within pretreated beech. For that purpose, primary addition of Zn(NO_3_)_2_ solution promoted the ion‐exchange between Na^+^ ions in the carboxyl groups of pretreated beech by Zn^2+^ ions. After the addition of 2‐methylimidazole (2‐MeIm), ZIF‐8 nanoparticles of ≈420 nm were uniformly and firmly deposited on the lumen surface via H‐bonding and electrostatic interactions resulting in the ZIF‐8/beech composite (Figure 2g,k; Figure S3a, Supporting Information). The formation of the composite rarely occurs without the pretreatment as exemplified in Figure S3b in the Supporting Information. Elemental mapping images of the ZIF‐8/beech composite revealed the homogeneous distribution of ZIF‐8 nanoparticles on the lumen surface of vessels, fibers, and rays as indicated by the uniform detection of zinc (green) across the entire bulk wood structure (Figure 2l). Conventional vacuum impregnation or filtration of pre‐synthesized ZIF‐8 favors the agglomeration of the ZIF‐8 particles at the outer surface of wood, which blocks the pathway into the inner part of the wood and limits the inner functionalization of the bulk sample (Figure S4, Supporting Information), thus emphasizing the impact and importance of the in situ growth treatment on the distribution of MOF crystals (Figure 2g–l). The FTIR spectra of the ZIF‐8/beech composite displayed the bands at 1146, 420 cm^−1^ and 758 cm^−1^ associated with C—N, Zn—N and out‐of‐plane bending of the 2‐MeIm ring of ZIF‐8, respectively (Figure 2m).^33^ The asymmetric peak of COO^−^ shifted from 1593 cm^−1^ in the case of pretreated beech to 1581 cm^−1^ for the ZIF‐8/beech composite, owing to the changes in the metal–carboxylate interaction from COO^−^—Na^+^ to COO^−^—Zn^2+^. In addition, the XRD pattern of the ZIF‐8/beech composite revealed two broad diffraction peaks centered around 2*θ* = 16.1° and 22.3° associated with cellulose, along with the characteristic diffraction peaks at 2*θ* = 7.3°, 10.4°, 12.8°, 14.7°, and 18.1° corresponding to the (110), (200), (211), (220), and (222) crystal faces of ZIF‐8, thus confirming the successful formation of ZIF‐8 crystals (Figure 2n).

The presented protocol mainly differs from state‐of‐the‐art preparation methods in the pretreatment step. In the case of cellulose‐derived composites, the pretreatment involves TEMPO (2,2,6,6‐tetramethylpiperidine‐1‐oxyl radical)‐ mediated oxidation, esterification, cysteamine, or carboxymethylation reactions, which are performed in the presence of toxic chemicals such as sodium hypochlorite (NaClO), cyanuric chloride (C_3_Cl_3_N_3_), and sodium chloroacetate (ClCH_2_COONa).^34^ In contrast, the treatment with NaOH offers a simple and renewable solution for the targeted wood functionalization. To compare both processes, a conventional carboxymethylation process was also applied (sample code: ZIF‐8/CM beech). The results showed that both methods led to similar MOF distributions (Figure S5, Supporting Information).

Following the synthesis of the ZIF‐8/beech composite, a sorbent activation procedure was conducted. It involves two steps: washing with methanol followed by heating under vacuum to fully remove the excess precursors that could otherwise block the microporous structure of ZIF‐8 (Figure S6, Supporting Information).^35^ As exemplified in Figure S7 in the Supporting Information, traces of the precursor were still evidenced in the composite when water was used as a solvent due to the lower solubility of 2‐MeIm in water compared to methanol, which suggests that the selected solvent has a key impact on the final porosity and morphological properties of the composite.

**Figure 3:**
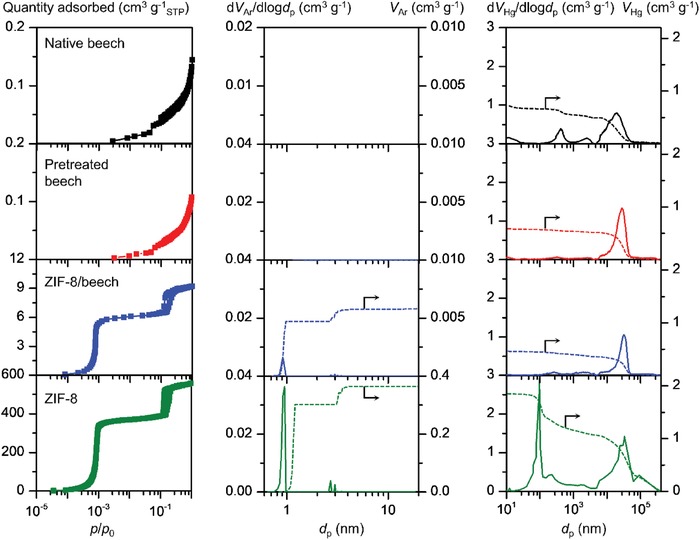
Adsorption isotherms (left), pore size and cumulative pore volumes from argon sorption (middle) and mercury porosimetry (right) of native beech, pretreated beech, ZIF‐8/beech composite and pure ZIF‐8.

In order to determine the complete distribution of macropores, mesopores, and micropores of the ZIF‐8/beech composite, argon sorption, and mercury porosimetry analyses were conducted (**Figure** **3**). The argon adsorption isotherms of both native and pretreated beech revealed negligible porosity whereas the resulting ZIF‐8/beech composite clearly exhibited the characteristic features of the ZIF‐8 bulk material, i.e., the presence of microporosity along with the hysteresis loop, which is associated with capillary condensation taking place in mesopores. The porous properties derived from the adsorption isotherms are summarized in **Table** **1**. The ZIF‐8/beech composite exhibited a surface area of 26 m^2^ g^−1^, which is 130 times higher than that obtained for native beech, and a micropore volume of 0.006 cm^3^ g^−1^. The substantial increase of the surface area compared to native beech proves the existence of ZIF‐8 within wood, in line with previous analyses (Figure 2g–n). Indeed, the comparison of the porous properties of native beech and the composite revealed a ZIF‐8 loading of ≈1.8 wt.% in the composite. The MOF loading could be further increased by longer MOF synthesis times, by layer‐by‐layer buildup of MOF multilayers or simply by using wood species with higher porosity. Increasing the synthesis time up to 48 h resulted in a surface area of 39 m^2^ g^−1^ and by using the two cycles layer‐by‐layer method during the ZIF‐8 synthesis, the surface area of the composite reached values up to 84 m^2^ g^−1^, which is 419.5 times higher than that obtained for native beech (Table S2, Supporting Information). The application of the nonlocal density functional theory (NLDFT) to the argon sorption isotherms enabled to determine the pore size distribution (PSD) in the micro and mesopore ranges (Figure 3, middle panel). Similarly, the PSD in the macropore range was calculated from the mercury intrusion data (Figure 3, right panel). The results revealed the absence of micro‐ and mesoporosity for the native and pretreated beech and the presence of micropores of ≈0.8 nm and mesopores of ≈3 nm in both the composite and pure ZIF‐8, which confirms the successful incorporation of ZIF‐8 into the wood matrix. The PSD obtained from the mercury porosimetry data of native wood evidenced the presence of pores of 10 nm, 400 nm, 0.3 µm and 20 µm, from which the first three diminished during the pretreatment step, as conventional vacuum drying after the pretreatment leads to the shrinkage of the cell walls. Therefore, the pores diminish within the cell walls and pits, as well as the lumen of the fibers and vessels. Bigger pores are preserved upon incorporation of ZIF‐8, thus suggesting the trimodal porosity of the final ZIF‐8/beech composite. The macropores (100 nm and 100 µm) detected in pure ZIF‐8 originate from the intraparticle porosity between the nanocrystals, and therefore, were not detected in the case of the composite owing to the uniform monolayer distribution of ZIF‐8 on wood. Accordingly, the final material evidenced a multimodal porous structure consisting of interconnected pores with different lengths ranging from micro‐ and meso‐ to macropores and can be accurately tuned by modifications in the synthesis protocol and/or in the selected MOFs and wood species.

**Table 1:** Porous properties of native beech, pretreated beech, ZIF‐8/beech composite, and pure ZIF‐8

Sample	*S*_BET_ [m^2^ g^−1^]^a)^	*S*_meso_ [m^2^ g^−1^]^b)^	*V*_micro_ [cm^3^ g^−1^]^c)^	*V*_pore_ [cm^3^ g^−1^]^c)^
Native beech	0.2	0.2	0	0
Pretreated beech	0.1	0.1	0	0
ZIF‐8/beech	26	4	0.006	0.011
ZIF‐8	1562	222	0.39	0.68

^a)^ BET method

^b)^ *t*‐plot method

^c)^ Volume adsorbed at *p/p_0_* = 0.99.

This type of hierarchically‐organized porous materials is of high relevance for applications dealing with large molecules and nanomaterials. The microporous network of the composite provides the shape selectivity function for guest molecules such as CO_2_ and N_2_ while the meso‐ and macroporous networks can improve the accessibility to the active sites and the diffusion kinetics. To exemplify the functionality of the composite, we have evaluated the adsorption capacity of CO_2_ and compared it with that of pure ZIF‐8 (Figure S8b, Supporting Information). The amount of CO_2_ adsorbed per gram of ZIF‐8 in the case of the composite (≈48 cm^3^ g^−1^_ZIF‐8_ at *p*/*p*_0_ = 1) is comparable to that of pure ZIF‐8 (≈44 cm^3^ g^−1^_ZIF‐8_ at *p*/*p*_0_ = 1) in line with the similar crystal sizes, i.e., 420±113 and 460±57 nm for ZIF‐8 within the composite and pure ZIF‐8, respectively. This indicates (i) the efficient utilization of ZIF‐8 in the composite and (ii) that the embedding process did not alter the adsorption capacity of the MOF. The adsorption capacity of N_2_ was also evaluated under equivalent experimental conditions. The composite did not adsorb N_2_ and adsorbs CO_2_ for two reasons. On the one hand, the pore aperture size and the affinity of imidazolate linker for CO_2_ influence the adsorption capacity of ZIF‐8 to CO_2_/N_2_.^36^ In detail, ZIF‐8 possesses cavities with narrow apertures (3.4 Å), which are close to the kinetic diameter of CO_2_ (3.3 Å), but smaller than that of N_2_ (3.64 Å), so that ZIF‐8 exhibit a stringent molecular sieving capability for CO_2_/N_2_ separation by allowing the transport of CO_2_ while blocking the passage of N_2_.^37^ Additionally, the existence of amine groups within imidazoleate linker enhances the adsorption of CO_2_.^38^ On the other hand, wood itself possesses carboxyl groups that enable the adsorption of CO_2_, but not of nitrogen.^39^ The results demonstrate the superior selectivity of the composite towards CO_2_ adsorption.

**Figure 4:**
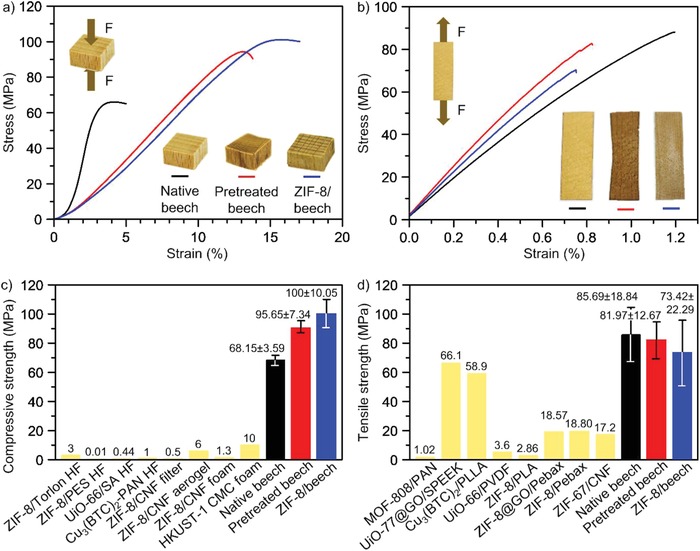
Mechanical performance of native beech, pretreated beech and ZIF‐8/beech composite. a) Compressive and b) tensile stress−strain curves of native beech, pretreated beech and ZIF‐8/beech composite. The insets in (a) and (b) show the schematic representation of the respective mechanical tests and the images of native beech, pretreated beech and ZIF‐8/beech composite samples used in the tests. Comparison of c) compressive strength and d) ultimate tensile stress of the materials used in this work with other polymer substrates/templates‐supported MOF composites (additional details are provided in Tables S3 and S4 in the Supporting Information).

In addition to the functionality, mechanical properties are key for further processing and application of the final composite. Upon pressure‐driven applications in gas adsorption and separation processes, the MOF/wood composites need to be strong enough to retain the hierarchical porosity under the applied pressure conditions. Therefore, we have conducted a mechanical characterization by compression and tensile tests (**Figure** **4**a,b; Figure S9, Supporting Information). Compression tests in the longitudinal direction of wood showed that pretreated beech exhibited higher strength (96 MPa) than native beech (68 MPa), but experienced pronounced reduction of the elastic modulus in compression and showed a threefold higher strain‐to‐failure value. Wood became slightly more flexible after the pretreatment step owing to the partial removal of the cell wall matrix components, lignin and hemicellulose (Figure 4a; Figure S9a, Supporting Information). The compressive strength of the ZIF‐8/beech composite was 100 MPa and showed similar mechanical performance as the pretreated beech, thus demonstrating that the presence of ZIF‐8 crystals within wood did not significantly affect the mechanical properties. In tensile tests in the longitudinal direction, native beech, pretreated beech and the ZIF‐8/beech composites exhibited rather similar ultimate tensile stress levels, i.e., mean values 87, 81, and 73 MPa, respectively (Figure 4b; Figure S9b, Supporting Information).The stress–strain curves show that the pretreatment did not affect the tensile modulus, which indicates that the stress transfer between fibers was not altered. Notably, wood offers a robust scaffold, which supplies excellent mechanical properties to the final composite. Indeed, both compressive strengths and ultimate tensile stress of the ZIF‐8/beech composite reported in this study are substantially higher than those obtained for the widely‐synthesized polymer‐based MOF composites previously reported (Figure 4c,d; Tables S3 and S4, Supporting Information).

**Figure 5:**
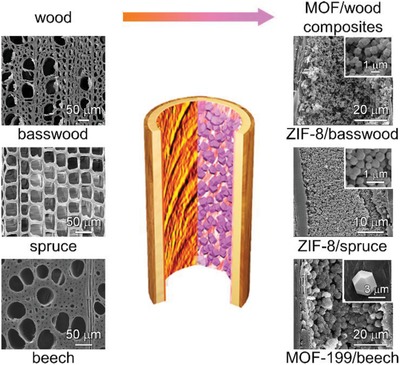
Schematic representation of the versatility of the synthetic approach to other wood species and MOFs. Cross‐sectional SEM images (left) of native wood (basswood, spruce and beech) and tangential‐sectional SEM images (right) of MOF/wood composites (ZIF‐8/basswood, ZIF‐8/spruce and MOF‐199/beech). The inset corresponds to the magnified SEM images of MOF nanocrystals in MOF/wood composites.

Finally, as a proof of concept, we demonstrate the feasibility of the synthesis protocol to be extended to other wood species, metal organic frameworks as well as different sample geometries depending on the final application. The developed synthesis approach was applied to MOF‐199 and other wood types, i.e., basswood and spruce. Both SEM (**Figure** **5**) and XRD (Figure S10, Supporting Information) analyses confirmed the successful formation of MOF/wood composites in all cases. The size of the ZIF‐8 particles in beech, spruce and basswood with different porosity (48%, 68%, and 78%) was 420 nm, 450 nm, and 1 µm respectively,^40^ owing to the different impregnation and transport behavior within different porous wood species. Additionally, MOF/wood composite can be built in different shapes. Indeed, ZIF‐8 nanocrystals were successfully synthesized within wood materials with different thicknesses and widths, defined as tangential cut beech samples and cuboid beech samples, and their morphologies and surface areas were similar (Figure S11, Supporting Information). These results clearly reveal the high potential of wood‐derived composites towards the practical implementation of MOFs.

## 3 Conclusion

In this work, we have developed a universal strategy for the green synthesis of a ZIF‐8/beech composite by the in situ growth of ZIF‐8 nanocrystals within beech. The effective growth of ZIF‐8 was facilitated by the pretreatment of beech with a sodium hydroxide solution. The as‐synthesized hierarchical ZIF‐8/beech composite exhibits a complex network of macropores, mesopores, and micropores resulting in a 130‐times higher surface area compared to native beech. Assessment of the CO_2_ adsorption capacity revealed the efficient utilization of the ZIF‐8 loading along with the preservation of the adsorption ability upon embedding of ZIF‐8 into the composite. Most importantly, this composite demonstrates excellent mechanical properties. Evaluation of the compressive strength and ultimate tensile stress resulted in values of 100 and 74 MPa, respectively, which surpass those obtained with state‐of‐the‐art polymer‐derived composites. In addition, the versatility of this synthetic approach was further proved with other MOFs, i.e., MOF‐199, and wood types, i.e., basswood and spruce, thus evidencing the wide potential for tuning the properties of the final material for a targeted application. The reported functionalization strategy offers a low‐cost, sustainable, and scalable platform with great potential in the fabrication of functional materials.

## 4 Experimental Section

##### Materials

European beech (*Fagus sylvatica*), American basswood (*Tilia americana*), and Norway spruce (*Picea abies*) wood samples were cut into cuboids with the dimensions 10 × 10 × 5 mm^3^ (radial × tangential × longitudinal, *R* × *T* × *L*). Tangential cut beech samples with a thickness of 6 mm, a length of 6 cm, and a width of 2 cm were used. Zinc nitrate hexahydrate (Zn(NO_3_)_2_·6H_2_0, 98%), copper(II) acetate (Cu(OAc)_2_, 98%), triethylamine (C_3_H_9_N, ≥99%), and sodium chloroacetate (ClCH_2_COONa, 98%) were purchased from Sigma‐Aldrich. 2‐Methylimidazole (2‐MeIm, C_6_H_6_N_2_, 97%) and sodium hydroxide (NaOH) were supplied by Thermo Fisher GmbH. 1,3,5‐benzenetricarboxylic acid was obtained from EMD Millipore Corporation. Methanol (≥99.9%), ethanol (≥99.8%), and *N*,*N*‐dimethyformamide (DMF, ≥99.8%, Sigma‐Aldrich) were used as solvents. All chemicals were used as received.

##### Wood Pretreatment

Native beech wood samples were pretreated by immersion in a 15% w/v NaOH aqueous solution for 1 h. The resulting wood samples denoted as pretreated beech, were washed under stirring with water for 24 h until the pH value of the washing solution reached 9.

##### Preparation of ZIF‐8/beech Composite

The pretreated beech wood samples were vacuumed for 1 h prior to the vacuum impregnation with a Zn(NO_3_)_2_ solution, which was prepared by dissolving Zn(NO_3_)_2_·6H_2_0 (2.4 g, 0.002 mol) in methanol (20 g) and deionized water (3 g). The impregnation time was 2 h to ensure a sufficient ion‐exchange between Zn and Na ions.

A 2‐MeIm solution, containing 13.2 g (0.04 mol) of MeIm in methanol (20 g) and deionized water (3 g) was subsequently added to the above solution. Stirring at room temperature for 24 h led to the ZIF‐8/beech composite. The composite was then rinsed three times with 50 mL of methanol for 5 min to remove the unreacted precursors, followed by drying in the vacuum‐oven at 103 °C for 48 h. The same synthesis procedure was used for the other wood species. For the preparation of the two cycles layer‐by‐layer sample, the above‐described synthesis procedure was repeated twice.

##### Preparation of MOF‐199/beech Composite

The synthesis route of MOF‐199 was adapted from a method reported by Tranchemontagne et al.^41^ Similarly to the procedure used in the preparation of the ZIF‐8/beech composite, a vacuum impregnation protocol was applied. After the pretreated beech was vacuumed for 1 h, a Cu(OAc)_2_ solution prepared by dissolving Cu(OAc)_2_ (860 mg) in a DMF:ethanol:water (12 ml, 1:1:1, v/v/v) solution was added. The resulting mixture was stirred for 24 h in order to ensure a sufficient ion‐exchange between Cu and Na ions. 500 mg of 1,3,5‐benzenetricarboxylic acid previously dissolved in 12 mL of the same solvent mixture were subsequently added dropwise followed by the addition of 0.5 mL of trimethylamine. The resulting mixture was stirred at room temperature and the final MOF‐199/beech composite was obtained after 24 h. The composite was rinsed three times with 25 mL DMF for 5 min to remove the unreacted precursors and then immersed in ethanol for 30 min before drying under vacuum at 103 °C for 48 h.

##### Materials Characterization

SEM was performed using a FEI Quanta 200F instrument. Prior to the analysis, a 10 nm thick Pt/Pd layer was sputtered onto all the sample surfaces to improve conductivity using a Safematic CCU‐010 Metal Sputter Coater. Energy dispersive X‐ray (EDX) spectroscopy was conducted using an Ametek‐EDAX Octane Super detector in secondary electrons mode at 20 kV and a working distance of ≈10 mm. AFM images were acquired in air using a NanoWizard 4 microscope (JPK Instruments AG – Bruker Nano GmbH). Specific measurement parameters are reported in the Supporting Information. ATR‐FTIR spectra were measured on a Bruker Tensor 27 spectrometer equipped with an ATR module. Wood samples used for testing were 0.1 g fibers cut from cuboids with a length of 5 mm and a width of 0.2 mm. Measurements were carried out in the spectral range from 650–4000 cm^−1^. Each measured spectrum was an average of 64 scans and the displayed spectrum is an average of 10 spectra. Baseline correction of the obtained average spectra was conducted in the software OPUS (Bruker). Zeta potential analysis was conducted using a SurPASS Electrokinetic Analyzer (Anton Paar). Specific measurement parameters and procedures are reported in the Supporting Information. Powder XRD was measured using a PANalytical X'Pert PRO MPD diffractometer with a Ni‐filtered CuKα radiation (λ = 0.15418 nm). For the measurements, small cut beech samples with a thickness of 6 mm, a length of 2 cm, and a width of 1 cm and MOF (ZIF‐8 and MOF‐199) powders were used. The diffraction data was recorded in the 2*θ* = 5°–60° range with an angular step size of 0.03° and a counting time of 1 s per step.

Ar sorption at −196 °C was undertaken in a Micromeritics 3Flex instrument. Prior to the measurement, the samples were evacuated at 90 °C for 48 h. Mercury intrusion porosimetry was conducted with a Micromeritics Autopore IV 9510 following in situ sample evacuation and using a contact angle of 140°. Adsorption isotherms of N_2_ and CO_2_ at 0 °C were recorded using a Micromeritics 3Flex Analyzer. Prior to the measurement, the samples were evacuated at 90 °C for 48 h.

Compression tests and tensile tests were performed using a universal testing machine (Zwick Roell) equipped with a 10 kN load cell. Prior to the compression tests, ten specimens with the dimensions of 10 × 10 × 5 mm^3^ (*R* × *T* × *L*) were dried in an oven at 65 °C until constant mass. The testing speed was 0.5 mm min^−1^ and a preloading of 100 N was used. For the tensile tests, ten previously dried specimens with the dimensions 6 × 20 × 60 mm^3^ (*R* × *T* × *L*) were used with 40 mm initial length between two grips. Due to the sample geometry, the term “ultimate tensile stress” was used instead of “tensile strength.” The preload was set to 10 N and the displacement was measured with a travel sensor at a speed of 0.35 mm min^−1^. Both the compression and tensile tests were conducted at 20 °C and 65% relative humidity.

## Conflict of Interest

The authors declare no conflict of interest.

## Author Contributions

K.T. and B.P. contributed equally to this work. K.T., B.P., J.P.R., and T.K. conceived the study. K.T., B.P., M.V‐A., Y.W., J.S., and J.T. performed experiments and analyzed data. K.T., B.P., I.B., J.P.R., and T.K. co‐wrote the manuscript. All authors discussed the results and commented on the manuscript.

## Supporting information

Supporting Information
