# Supplementary material for: Green Synthesis of Hierarchical Metal–Organic Framework/Wood Functional Composites with Superior Mechanical Properties
Source: Adv Sci (Weinh). 2020 Feb 6;7(7):1902897. doi: 10.1002/advs.201902897 (PMC7141016; doi:10.1002/advs.201902897)
Supplement: Supplementary file 1 — Supporting Information [file ADVS-7-1902897-s001.pdf]

Copyright WILEY-VCH Verlag GmbH & Co. KGaA, 69469 Weinheim, Germany, 2020.

ADVANCED SCIENCE
Open Access

Supporting Information

for *Adv. Sci.,* DOI: 10.1002/advs.201902897

Green Synthesis of Hierarchical Metal–Organic Framework/ Wood Functional Composites with Superior Mechanical Properties

Kunkun Tu, Begoña Puértolas, Maria Adobes-Vidal, Yaru Wang, Jianguo Sun, Jacqueline Traber, Ingo Burgert, Javier Pérez-Ramírez, and Tobias Keplinger**

Copyright WILEY-VCH Verlag GmbH & Co. KGaA, 69469 Weinheim, Germany, 2020.

Supporting Information

Green Synthesis of Hierarchical Metal-Organic Framework/Wood Functional Composites with Superior Mechanical Properties

Kunkun Tu, Begoña Puértolas, Maria Adobes Vidal, Yaru Wang, Jianguo Sun, Jacqueline Traber, Ingo Burgert, Javier Pérez-Ramírez, Tobias Keplinger

1. Experimental section

1.1. Materials synthesis

Preparation of carboxymethylated wood (CM wood)

Native beech was immersed in a 1 M sodium chloroacetate and 15% w/v NaOH solution for 1 h. The resulting CM beech was thoroughly washed with deionized water for 24 h in order to remove the residual reactants.

Preparation of ZIF-8/CM beech composite

The CM beech samples were vacuumed for 1 h prior to the vacuum impregnation with a $Zn(NO_3)_2$ solution, which was prepared by dissolving $Zn(NO_3)_2 \cdot 6H_20$ (2.4 g, 0.002 mol) in methanol (20 g) and deionized water (3 g). The impregnation time was 2 h to ensure a sufficient ion-exchange between Zn and Na ions at the surface of the lumen. A 2-MeIm solution, containing 13.2 g (0.04 mol) of MeIm in methanol (20 g) and deionized water (3 g) was subsequently added to the above solution. Stirring at room temperature for 24 h led to the ZIF-8/CM beech composite. The resulting material was then rinsed three times with 50 ml of methanol for 5 min to remove the unreacted precursors, followed by drying in the vacuum-oven at 103°C for 48 h.

Synthesis of ZIF-8

For the synthesis of ZIF-8, a solution of 2.4 g of $Zn(NO_3)_2 \cdot 6H_20$ in 20 g of methanol and 3 g of deionized water and a second a solution containing 13.2 g of 2-MeIm in the same solvent mixture were prepared. Both solutions were mixed and then stirred at room temperature for 24 h. The obtained solid was collected by centrifugation and thoroughly washed with methanol (3 times). The product was subsequently dried under vacuum overnight at 103°C. By adding 0.5 g of ZIF-8 powders into 50 ml of methanol, a 1% w/v ZIF-8 solution was formed.

Synthesis of MOF-199

860 mg of $Cu(OAc)_2$ were dissolved in a 12 ml solvent solution of DMF:ethanol:water (1:1:1). 500 mg of 1,3,5-benzenetricarboxylic acid were subsequently dissolved in 12 ml of the same solvent mixture. Both solutions were mixed and then stirred vigorously at room temperature for 24 h. The obtained solid was collected by centrifugation and thoroughly washed (3 times) with 30 ml of DMF, followed by drying under vacuum overnight at 103°C.

Preparation of ZIF-8/beech by vacuum impregnation method

Pretreated beech wood samples were vacuumed for 1 h before adding the 1% w/v ZIF-8 solution. The vacuum impregnation was sustained 24 h, after which the resulting ZIF-8/beech composite was dried in the vacuum-oven at 103°C for 48 h.

Preparation of ZIF-8/beech by filtration method

The filtration method is adapted from a protocol reported by Sun *et al.*[1] In short, a beech wood cube was placed with its cross section facing up into a suction filter. 10 ml of 1% w/v ZIF-8 solution were then slowly poured into the filter and soaked through by vacuum. The procedure was repeated 3 times followed by drying of the sample in the vacuum-oven at 103°C for 48 h.

1.2. Materials characterization

Zeta potential analysis was conducted using a SurPASS Electrokinetic Analyzer (Anton Paar). Tangential beech samples were cut into dimensions of 2×10×20 mm^3 (R×T×L) and attached to an adjustable gap cell (gap height was adjusted to 100 μm ± 20 μm) by double-sided adhesive tape. Prior to the measurements, native wood was conditioned by immersion in deionized water, and pretreated wood as well as CM wood were conditioned by immersion into a NaOH solution (pH 9) overnight to avoid swelling of the wood during the measurement. Measurements were carried out at 400 mbar in a 0.001 M KCl electrolyte solution at pH 5.7±0.1 (native wood) and pH 9 (pretreated wood and CM wood). The mean value and

standard deviation of the ζ potential for the native wood, pretreated wood and CM wood were obtained from eight independent ramps measured for two samples each.

Atomic force microscopy (AFM) images of native and pretreated beech wood were acquired in air and in solution using a NanoWizard 4 microscope (JPK Instruments AG - Bruker Nano GmbH). The beech cubes were glued to AFM sample mounting disks using cementit universal adhesive (Merz+Benteli ag) with the tangential section oriented parallel to the disk surface. The cube surface was then polished with a microtome equipped with a steel knife (RM2255, Leica) under wet conditions. Measurements in air were performed under controlled climatic conditions (65% R.H., 20°C) in alternating contact mode (AC mode), using rectangular-shaped silicon cantilevers (NCHR, Nano World) with a resonant frequency of 320 kHz and 42 N m^{-1} nominal spring constant. The images were obtained at a line scan rate of 0.4 Hz and a resolution of 512×512 pixels. Topographical investigations in solution were performed on cubes submerged in 5 ml of deionized water in contact mode (CM mode) using rectangular-shaped silicon cantilevers (CONT, Nano World) with a resonant frequency of 13 kHz and 0.2 N m^{-1} nominal spring constant. The images were obtained at line scan rates of 0.1-0.4 Hz, a setpoint of 0.5-2.5 nN and a resolution of 512×512 pixels. All images were first analyzed with the JPK image processing software (JPK Instruments AG), where a first order polynomial fit correction was applied to each scan line to correct large background height changes, and then plotted using Gwyddion 2.44.

2. Results section

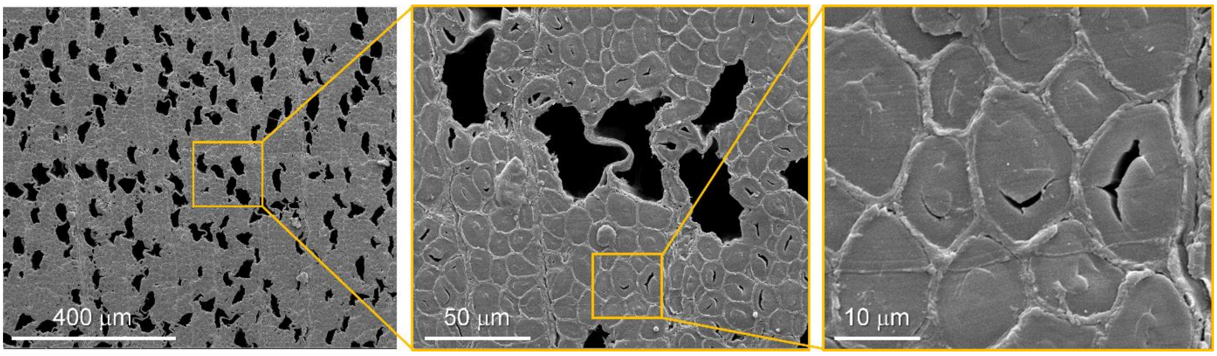

Figure S1. Cross-sectional SEM images at different magnification of beech after the NaOH pretreatment followed by a drying process at 103°C under vacuum.

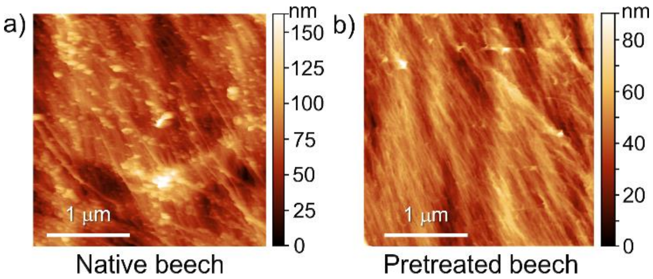

Figure S2. AFM images of a) native beech and b) pretreated beech tested under *in situ* contact mode in wet state.

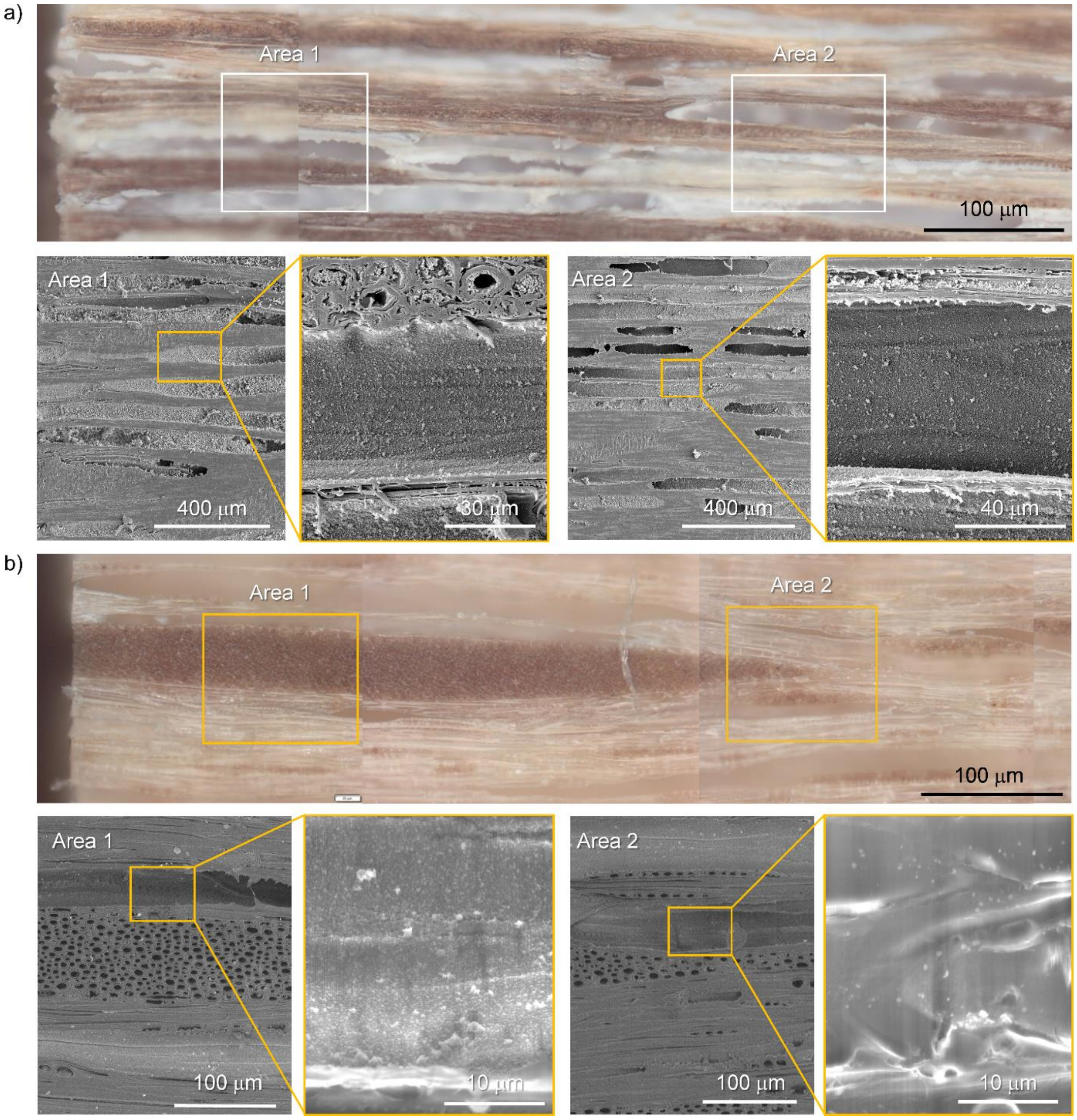

Figure S3. Light microscope and SEM images of ZIF-8/beech composites a) with and b) without NaOH pretreatment.

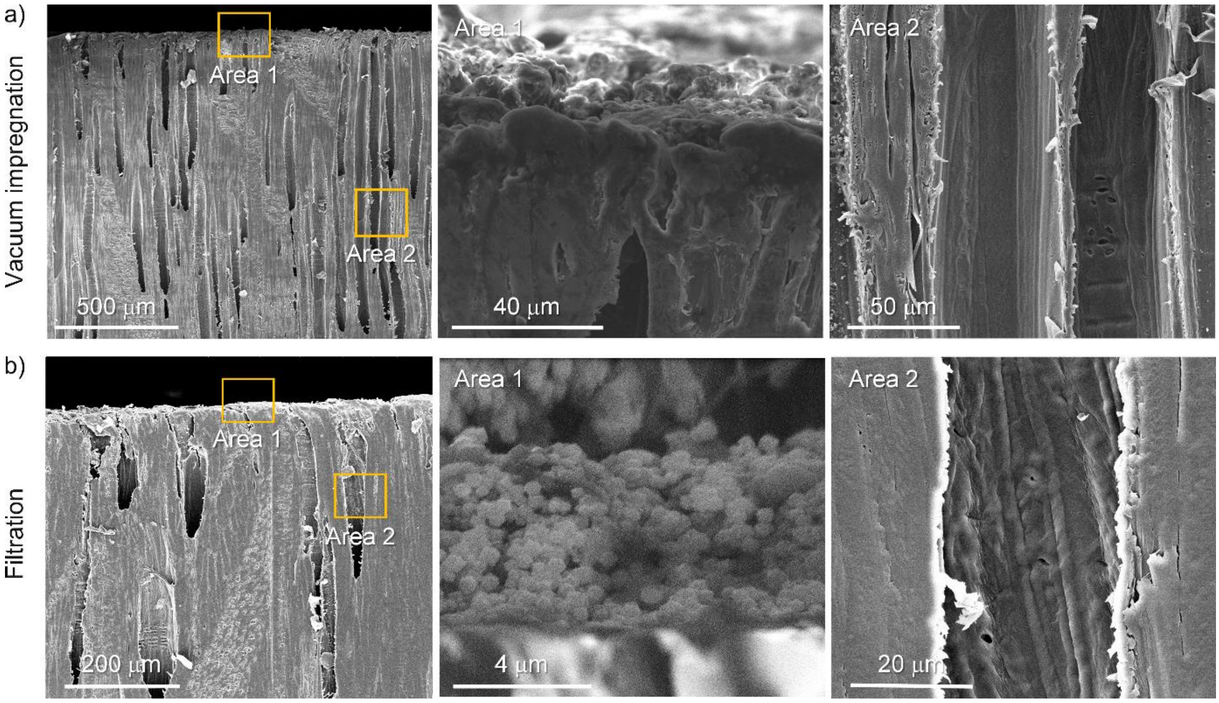

Figure S4. SEM images of the ZIF-8/beech composites fabricated by a) vacuum impregnation and b) filtration methods.

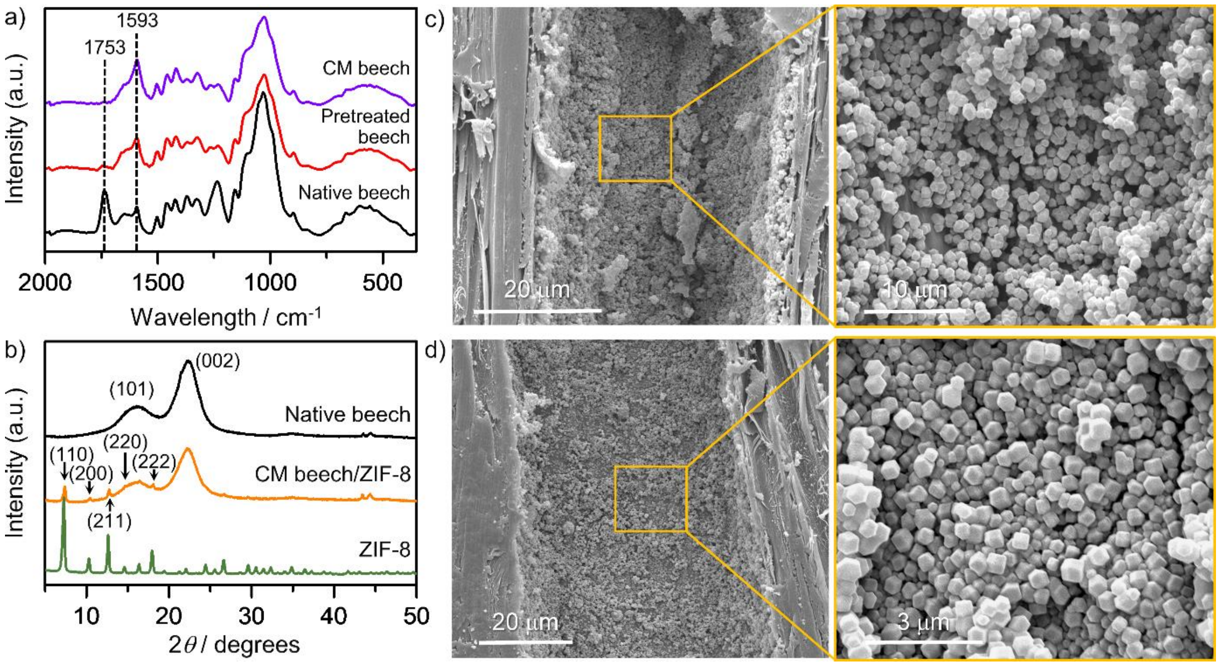

Figure S5. a) FTIR spectra of CM beech, pretreated beech and native beech. b) XRD patterns of native beech, ZIF-8/CM beech and pure ZIF-8. c) SEM images of ZIF-8/CM beech composite. d) SEM images of ZIF-8/beech composite.

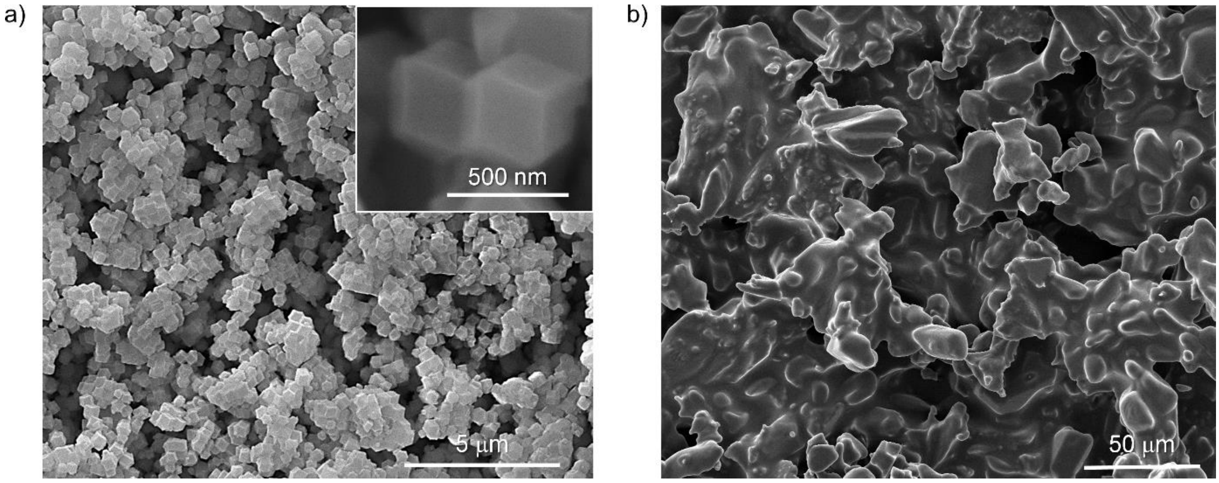

Figure S6. SEM images of pure ZIF-8 a) with and b) without washing with methanol after the synthesis process.

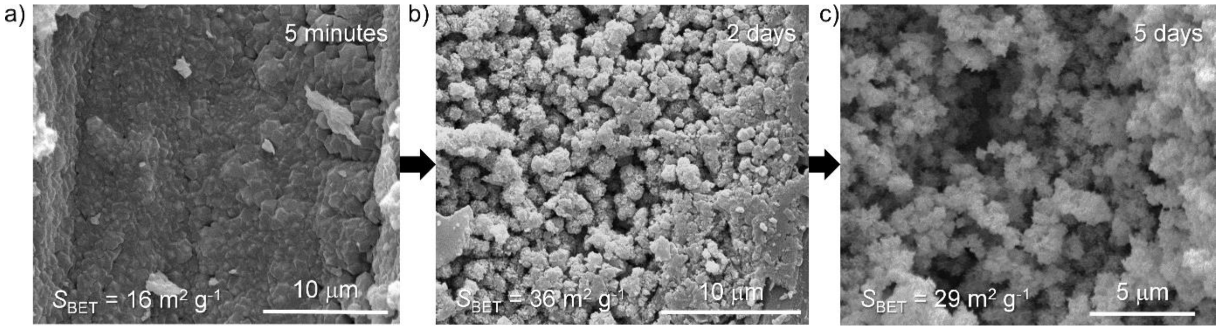

Figure S7. SEM images and surface areas of the ZIF-8/beech composites washed with water during a) 5 minutes, b) 2 days and c) 5 days.

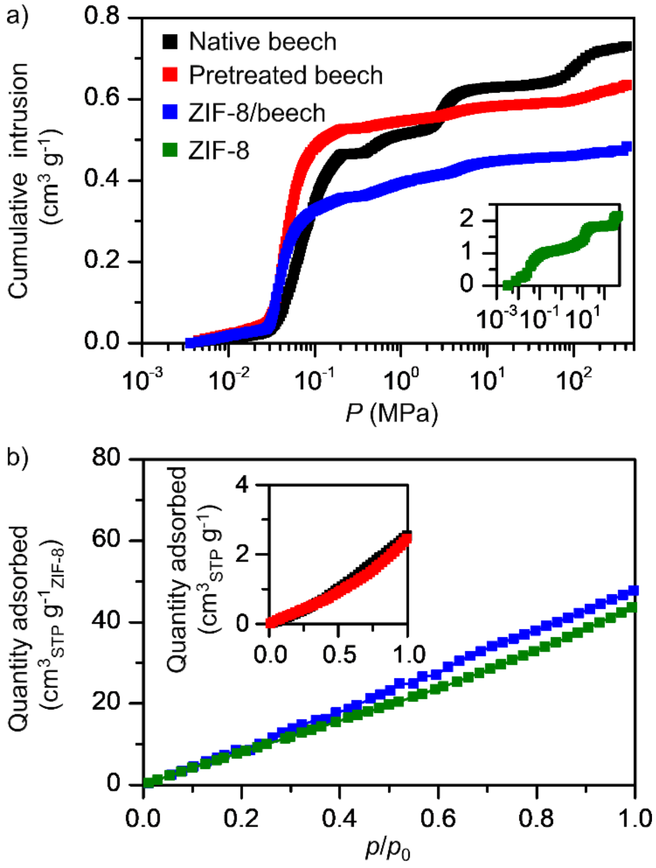

Figure S8 a) Mercury intrusion of native beech, pretreated beech and ZIF-8/beech composite. The inset represents the mercury intrusion of pure ZIF-8. b) CO_2 adsorption isotherms of ZIF-8/beech composite and pure ZIF-8 expressed per gram of ZIF-8. The inset corresponds to the adsorption isotherms of native beech and pretreated beech expressed per gram of sample.

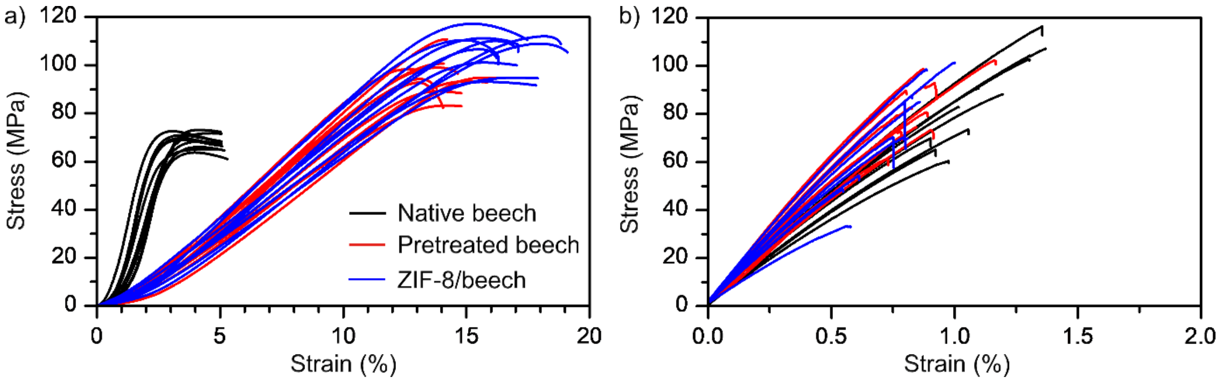

Figure S9. a) Compressive and b) tensile stress-strain curves of native beech, pretreated beech and ZIF-8/beech composite.

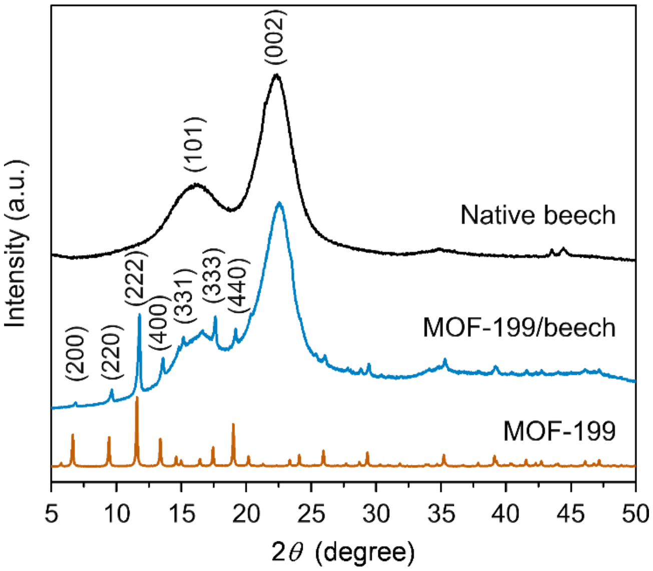

Figure S10. XRD patterns of native beech, MOF-199/beech composite and pure MOF-199.

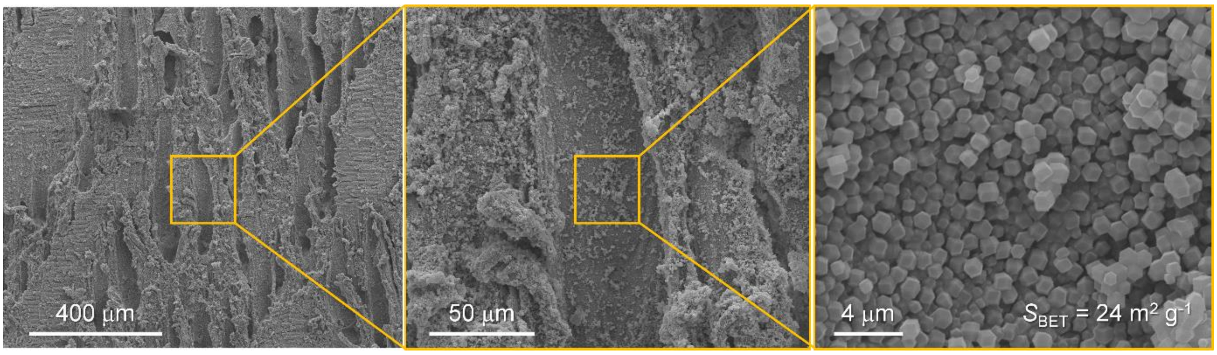

Figure S11. SEM images of ZIF-8/beech (tangential cut sample) at different magnification.

Table S1. Zeta potential of native beech, pretreated beech and CM beech.

Sample	Zeta potential [mV]
Native beech	-12.05±1.87
Pretreated beech	-38.44±1.99
CM beech	-64.24±1.29

Table S2. Porous properties of ZIF-8/beech composites prepared with different time and synthesis method.

Sample	S_{BET} [m^2 g^{-1}][a)]	S_{meso} [m^2 g^{-1}][b)]	V_{micro} [cm^3 g^{-1}][c)]	V_{pore} [cm^3 g^{-1}][c)]
ZIF-8/beech (1 h synthesis)	16	3	0.006	0.01
ZIF-8/beech (24 h synthesis)	26	4	0.006	0.011
ZIF-8/beech (48 h synthesis)	39	5.6	0.01	0.016
ZIF-8/beech (layer-by-layer)	84	14.6	0.02	0.037

[a)] BET method; [b)] *t*-plot method; [c)] Volume adsorbed at p/p_0=0.99.

Table S3. Comparison of the compressive strength of the ZIF-8/beech composite with other polymer substrates/templates-supported MOFs composites.

Material	Compressive strength [MPa]	Ref.
ZIF-8/Torlon HF	3	[2]
ZIF-8/PES HF	0.01	[3]
UiO-66/SA HF	0.44	[4]
$Cu_3(BTC)_2$-PAN HF	1	[5]
ZIF-8/CNF filter	0.5	[6]
ZIF-8 cellulose aerogel	6	[7]
ZIF-8 cellulose foam	1.3	[8]
HKUST-1 CMC foam	10	[9]
Native beech	68.15±3.59	This work
Pretreated beech	95.65±7.34	This work
ZIF-8/beech	100±10.05	This work

Table S4. Comparison of the ultimate tensile stress of the ZIF-8/beech composite with other polymer substrates/templates-supported MOFs composites.

Material	Tensile strength [MPa]	Ref.
MOF-808/PAN	1.02	[10]
UiO-66@GO/SPEEK	66.1	[11]
$Cu_3(BTC)_2$ /PLLA	58.9	[12]
UiO-66/PVDF	3.6	[13]
ZIF-8/PLA	2.86	[14]
ZIF-8@GO/Pebax	18.57	[15]
ZIF-8/Pebax	18.8	[15]
ZIF-67/CNF	17.2	[16]
Native beech	85.691±18.84	This work
Pretreated beech	81.97±12.67	This work
ZIF-8/beech	73.42±22.29	This work

References

[1] J. Sun, T. Yang, C. Wang, L. Chen, *Nano Energy* **2018**, *48*, 383.

[2] A. M. Marti, W. Wickramanayake, G. Dahe, A. Sekizkardes, T. L. Bank, D. P. Hopkinson, S. R. Venna, *ACS Appl. Mater. Interfaces* **2017**, *9*, 5678.

[3] P. Su, W. Li, C. Zhang, Q. Meng, C. Shen, G. Zhang, *J. Mater. Chem. A* **2015**, *3*, 20345.

[4] Y. Chen, F. Chen, S. Zhang, Y. Cai, S. Cao, S. Li, W. Zhao, S. Yuan, X. Feng, A. Cao, X. Ma, B. Wang, *J. Am. Chem. Soc.* **2017**, *139*, 16482.

[5] W. Li, Z. Yang, G. Zhang, Z. Fan, Q. Meng, C. Shen, C. Gao, *J. Mater. Chem. A* **2014**, *2*, 2110.

[6] S. Ma, M. Zhang, J. Nie, J. Tan, B. Yang, S. Song, *Carbohydr. Polym.* **2019**, *203*, 415.

[7] L. Zhu, L. Zong, X. Wu, M. Li, H. Wang, J. You, C. Li, *ACS Nano* **2018**, *12*, 4462.

[8] S. Ma, M. Zhang, J. Nie, J. Tan, S. Song, Y. Luo, *Carbohydr. Polym.* **2019**, *208*, 328.

[9] Y. Chen, X. Huang, S. Zhang, S. Li, S. Cao, X. Pei, J. Zhou, X. Feng, B. Wang, *J. Am. Chem. Soc.* **2016**, *138*, 10810.

[10] J. E. Efome, *Doctor Thesis*, University of Ottawa, June, **2018**.

[11] H. Sun, B. Tang, P. Wu, *ACS Appl. Mater. Interfaces* **2017**, *9*, 26077.

[12] A. Kathuria, M. G. Abiad, R. Auras, *Polymer* **2013**, *54*, 6979.

[13] R. Semino, J. C. Moreton, N. A. Ramsahye, S. M. Cohen, G. Maurin, *Chem. Sci.* **2018**, *9*, 315.

[14] X. Dai, X. Li, X. Wang, *Chem. Eng. J.* **2018**, *338*, 82.

[15] L. Dong, M. Chen, J. Li, D. Shi, W. Dong, X. Li, Y. Bai, *J. Membrane. Sci.* **2016**, *520*, 801.

[16] L. Qian, D. Lei, X. Duan, S. Zhang, W. Song, C. Hou, R. Tang, *Carbohydr. Polym.* **2018**, *192*, 44.
